# The Deubiquitinase USP39 Promotes ESCC Tumorigenesis Through Pre-mRNA Splicing of the mTORC2 Component Rictor

**DOI:** 10.3389/fonc.2021.667495

**Published:** 2021-05-26

**Authors:** Yuan Zhao, Huiwu Geng, Gang Liu, Qiang Ji, Xiaomin Cheng, Xinying Li, Wei Liu, Rick F. Thorne, Renquan Zhang, Xiaoying Liu

**Affiliations:** ^1^ Department of Thoracic Surgery, The First Affiliated Hospital, Anhui Medical University, Hefei, China; ^2^ Biology Department, School of Life Sciences, Anhui Medical University, Hefei, China; ^3^ Translational Research Institute of Henan Provincial People’s Hospital and People’s Hospital of Zhengzhou University, Molecular Pathology Centre, Academy of Medical Sciences, Zhengzhou University, Zhengzhou, China

**Keywords:** esophageal squamous cell carcinoma (ESCC), ubiquitin specific protease 39 (USP39), mammalian target of rapamycin (mTOR), RNA splicing, rictor

## Abstract

Spliceosomes are large RNA-protein molecular complexes which mediate splicing of pre-mRNA in eukaryotic cells. Their function is frequently altered in cancer, providing opportunities for novel therapeutic approaches. The ubiquitin specific protease 39 (USP39) is a highly conserved deubiquitylation family member that plays an essential role in pre-mRNA splicing where it serves to assemble the mature spliceosome complex. Previous studies have reported that USP39 acts in an oncogenic manner where it contributes to cancer progression and predicts poor prognosis in various human tumor types. Here we report that USP39 is differentially upregulated in human esophageal squamous cell carcinoma (ESCC) and its expression is significantly associated with clinicopathological characteristics including differentiation status and TNM stage. We found the USP39 upregulation was maintained in ESCC cell lines where it functioned to promote cancer cell growth *in vitro* and in xenografts. RNA-seq analyses identified that mTOR pathway activation was affected by shRNA-mediated silencing of USP39. Subsequent biochemical analyses demonstrated that USP39 regulates the activity of mTORC2 by selectively enhancing the splicing and maturation of Rictor mRNA, although not other key mTORC components. Together, our report proposes USP39 as a biomarker and oncogenic factor in ESCC, with a potential for targeting the USP39/mTOR2/Rictor axis as a therapeutic strategy. Furthermore, our study adds ESCC to the list of cancers where USP39 contributes to tumorigenesis and progression.

## Introduction

With an increasing incidence worldwide, esophageal cancer represents one of the most aggressive and fatal cancers (1, 2). In the highest-risk areas, 90% of cases are esophageal squamous cell carcinoma (ESCC), the major histopathological type of this disease. Despite advances in treatment, five-year survival rates are less than 20% and these poor outcomes are associated with late stage diagnosis, frequent metastasis and therapeutic resistance (3, 4). Therefore, ESCC research is focused on understanding the molecular mechanisms governing disease initiation and progression with the goal of identifying novel therapeutic targets.

Spliceosomes are large RNA-protein molecular complexes which mediate splicing of pre-mRNA in eukaryotic cells. Major spliceosome components consist of different combinations of small nuclear RNAs (snRNAs) designated U1, U2, U4, U5 and U6 which form stable ribonuclear protein complexes (snRNPs), each involved at different stages of the splicing process. Other accessory proteins including recruiting factors such as the serine and arginine-rich (SR) proteins are also involved (5). The genes encoding spliceosome assembly components such as SF3B1, ZRSR2, U2AF1 and SRSF2 have been found to have frequent and recurrent somatic mutations in various types of cancers whereas other components may be overexpressed, collectively suggesting roles in oncogenesis (6–10). Notably, knockdown of spliceosome-related genes results in growth inhibition of breast, lung, and melanoma cancer cells, but has had little effect on the survival of the normal epithelial cells (11). Therefore, studies on the spliceosome and its related proteins may offer novel opportunities to enhance the efficacy of cancer therapies.

The ubiquitin specific protease 39 (USP39) is a highly conserved deubiquitylation family member (also known as Sad1p in yeast) and component of the U4/U6-U5 tri-snRNP. Each member of USPs contains a zinc finger ubiquitin binding domain and a ubiquitin C-terminal hydrolase (UCH) domain (12) although USP39 is notably deprived of ubiquitin protease activity due to the absence of three active site residues in its UCH-domain (13). USP39 plays an essential role in pre-mRNA splicing with studies showing it functions in the assembly of mature spliceosome complex, although not in the maintenance of complex stability (12). In particular, USP39 was shown to be involved in the splicing of Aurora B and other mRNAs essential for proper spindle checkpoint function (14). Consistently, mutation of zebrafish USP39 induces Rb1 splicing defects which induces G1/S arrest (15). Moreover, other studies have suggested that USP39 functions as an oncogenic factor in numerous cancers including breast (16), liver (17), medullary thyroid (18), lung (19), prostate (20), oral squamous cell (21) and renal cell carcinomas (22). For example, USP39 promotes prostate cancer cell tumorigenesis by facilitating EGFR mRNA maturation and transcriptional elongation (20), whereas in glioma it promotes progression by facilitating TAZ mRNA maturation (23). Our previous research in melanoma also implicated USP39 in disease progression through regulating cell cycle and apoptosis *via* ERK1/2 signaling (24).

To our knowledge, USP39 has not yet been previously investigated in ESCC. Here we report that USP39 is differentially upregulated in human ESCC compared with adjacent normal tissues and find that its expression is significantly associated with clinicopathological characteristics. We also found US39 upregulation was maintained in ESCC cell lines and showed it impacted measures of tumorigenesis both *in vitro* as well as xenograft experiments. We further demonstrate this oncogenic activity is due to USP39’s role in regulating the maturation of Rictor mRNA. Our data define USP39 as a biomarker and oncogenic factor in ESCC, with a potential for targeting USP39/mTOR2/Rictor pathway as a therapeutic strategy.

## Materials and Methods

### Cell Culture

The human ESCC cell line ECA109 was obtained from the Culture Collection of the Chinese Academy of Sciences (Shanghai, China); ESCC cell lines KYSE30, KYSE70, TE13 and the normal esophageal epithelial cell line Het-1A were all obtained from Kebai Biological Technology (Nanjing, China). Cells were cultured in RPMI 1640 medium (Gibco BRL, Rockville, MD, USA) with 5% fetal bovine serum, 100 U/ml of penicillin and 100 mg/ml streptomycin. Primary cultures of esophageal epithelial cells were prepared by modification of established methods. All cells were maintained at 37°C in a humidified chamber containing 5% CO_2_.

### Datasets

To investigate the clinical significance of USP39 in ESCC, we retrieved and analyzed the expression of USP39 in ESCC tissues and adjacent normal tissues using published data from public Gene Expression Omnibus (GEO) database (http://www.ncbi.nlm.nih.gov/geo) and normalized using Robust Multichip Average (RMA). Subsequently, we also acquired gene expression data from TCGA (https://portal.gdc.cancer.gov/projects/TCGA-LIHC) and analyzed the relationship between USP39 expression and disease-free survival.

### Clinical Specimens and Ethics

Fresh tumor tissues and paired adjacent normal tissues were collected from patients who underwent radical esophagectomy at the Department of Thoracic Surgery, the First Affiliated Hospital of Anhui Medical University between March 2014 and January 2017. Tissue samples were fixed in formalin and embedded in paraffin (FFPE). The patient’s clinicopathological characteristics were obtained from surgical and pathological records. The tumor stage utilized in the present study was according to the 8th edition of the American Joint Committee on Cancer (AJCC) TNM classification system. Patients received neither chemotherapy nor radiotherapy before resection. All human tumor tissues were obtained with written informed consent with a signature from patients or their guardians prior to participation in the study. This study was approved by the institutional review board of Anhui Medical University (20190402).

### Immunohistochemistry

Deparaffinized and hydrated FFPE tissue sections (5 μm) were subject to antigen retrieval using microwaving at 130°C for 10 min. Sections were then incubated for 15 min in 3% H_2_O_2_ to eliminate endogenous peroxidase before subsequent incubation with USP39 primary antibodies (1:100; Abcam, ab131332) overnight at 4°C. Secondary antibody incubations were performed at 37°C for 30 min, washed with three times with phosphate-buffered saline (PBS) for 5 min, then incubated with DAB and hematoxylin. Immunoreactivity was blindly evaluated by two professional pathologists to according to immunoreactivity score (IRS) system, which is based on the proportion and intensity of positively stained cells. The percentage of positive cells was scored as 0-4 where positive stained cells ≤5% (0), 6–25% (1), 26–50% (2), 51–75% (3), and >75% (4); while the intensity of staining was scored as 0-3: colorless (0), bright yellow (1), yellow (2), and brown (3). The overall staining scores were then calculated by multiplying the percentage and intensity staining scores. The staining score index was designated as negative (-, 0), weak positive (+, 1-4), moderate positive (++, 5-8), and strong positive (+++, 9-12). For analysis, we combined weak positive and negative staining cases as low USP39 expression, and moderate and strong positive staining as high USP39 expression.

### Lentiviral Transduction

The lentivirus particles containing shRNA constructs targeting USP39 (sh-USP39-1, shUSP39-2) or negative control (sh-control) were obtained from GeneChem (Shanghai, China). Transduction of ECA109 and KYSE30 cells was performed in the presence of polybrene (Sigma, USA) at a final concentration of 5 µg/ml according to the manufacturer’s instructions. Two days after transduction, GFP fluorescence was confirmed and cells further selected with 4-6 µg/ml puromycin. The sequences of the shRNAs and negative control used are as follow: sh-control, 5′-TTCTCCGAACGTGTCACGT-3′; sh-USP39-1, 5′-TTTGGAAGAGGCGAGATAA-3′; sh-USP39-2, 5′-CAAGTTGCCTCCATATCTA-3′.

### Plasmid Vectors and Transfection

A pcDNA3.1-based expression plasmid was constructed containing HA epitope-tagged USP39 cDNA and used to transfect ECA109 and KYSE30 cells using Lipofectamine 2000 reagent in Opti-MEM medium for 6h. The medium was replaced with fresh complete medium before conducting the indicated experiments.

### Quantitative Real-Time PCR (qRT-PCR)

Total RNA was extracted from the cells using Trizol reagent (Invitrogen, USA). Isolated total RNA was quantified and used to generate cDNA using a reverse transcription kit (Toyobo; Osaka, Japan). The mRNA levels were quantified by real-time PCR. The data were interpreted using the 2^-ΔΔCt^ method with GAPDH serving as a reference gene for normalization. All experiments were repeated at least 3 times. The primers used are shown in **Table 4**.

### Western Blotting Analysis

Total cell lysates were resolved on 12% denaturing polyacrylamide gels and electro-transferred to PVDF membranes. Membranes were blocked in TBST solution containing 5% (w/v) fat-free dry milk followed by triple washing in TBST and incubation with primary antibodies against human USP39 (#ab131332, abcam), mTOR (#2972s, CST), Raptor (20984-1-AP, proteintech), Rictor (#53A2, CST), Akt (#2920, CST), p-Akt (Thr308) (#9275s, CST), p-Akt (Ser473) (#4060, CST), S6K1 (#2217s, CST), p-Rps6 (#5364s, CST) or GAPDH (#60004-1-IG, proteintech), respectively, overnight at 4°. After washing three times, the membranes were incubated with horseradish peroxidase-conjugated secondary antibodies for 1h at room temperature. Proteins were detected using ECL reagent (Pierce, USA) and band intensities were normalized to GAPDH. Each experiment was performed for three times.

### Cell Proliferation Assay

Cells were plated into 96-well culture plates and after overnight culture, CCK-8 solution (Dojindo; Kumamoto, Japan) was added to evaluate cell proliferation every 24 hours for 4 consecutive days. The absorbance of the samples was measured at 450 nm in a microplate reader. Experiments were repeated at least three times.

### Colony Formation Assay

Cells were seeded at 1000 cells/well in 6-well plates and cultured at 37°C. Twelve days later, cells were washed twice with PBS, fixed with 4% formaldehyde, stained with 0.5% crystal violet for 10 min and washed with ddH_2_O for three times. Colonies were photographed with a digital camera.

### RNA-Seq Analysis

Total RNA from ECA109 cells (triplicates) bearing control or USP39 shRNA was extracted by the Qiagen RNeasy kit (Qiagen, Cat #74104). Libraries were prepared using VAHTS^®^ Total RNA-seq (H/M/R) Library Prep Kit (Vazyme) according to the manufacturer’s instructions. Samples were sequenced on an Illumina HiSeq6000 with paired-end 150 bp reads. Reads were then filtered for adapter contamination using cutadapt and so that at least 90% of bases of each read had a quality score >20. Reads were aligned to the reference genome (hg19) using STAR version 2.5.2b retaining only primary alignments. Reads overlapping blacklisted regions of the genome were then removed. Transcript abundance was then estimated using salmon, and differential expression was detected using DESeq2. Pathway analyses were then performed on significantly upregulated and significantly downregulated gene sets independently according to the KEGG (Kyoto Encyclopedia of Genes and Genomes) database (25).

### RNA Immunoprecipitation (RIP) Assay

RIP analysis was performed using the Magna RIP RNA-binding protein immunoprecipitation kit (Millipore, Billerica, MA, USA) and the USP39 antibody (#ab131332, Abcam) following the manufacturer’s protocol. Co-precipitated RNAs were isolated, purified, and subjected to qRT-PCR analysis.

### Animal Studies

Male BALB/c nude mice (at age 3-4 weeks; GemPharmatech Co., Ltd; Nanjing, China) were randomly divided into the indicated groups. ECA109 cells (6×10^6^) with or without USP39 shRNA knockdown were injected subcutaneously into the right flank of each mouse. Tumor sizes were measured by calipers every 3 days. Mice were killed and tumors were weighed at 28 days after tumor cell transplantation. Tumors were collected and either immediately snap-frozen and preserved at -80°C, or fixed in 10% formalin for 48 h before paraffin embedding and sectioning. Animal studies were also approved by the Animal Research Ethics Committee of Anhui Medical University.

### Statistical Analysis

Statistical analysis was performed using the SPSS 17.0 software package. Differences in categorical variables between two groups were analyzed by Chi-square test or Mann-Whitney U test. Analysis of variance and Student’s t test were used for comparison among different treatment groups. *P* < 0.05 was considered statistically significant. All experiments were carried out with at least three replicates.

## Results

### USP39 Is Upregulated in ESCC

**Figure 1:**
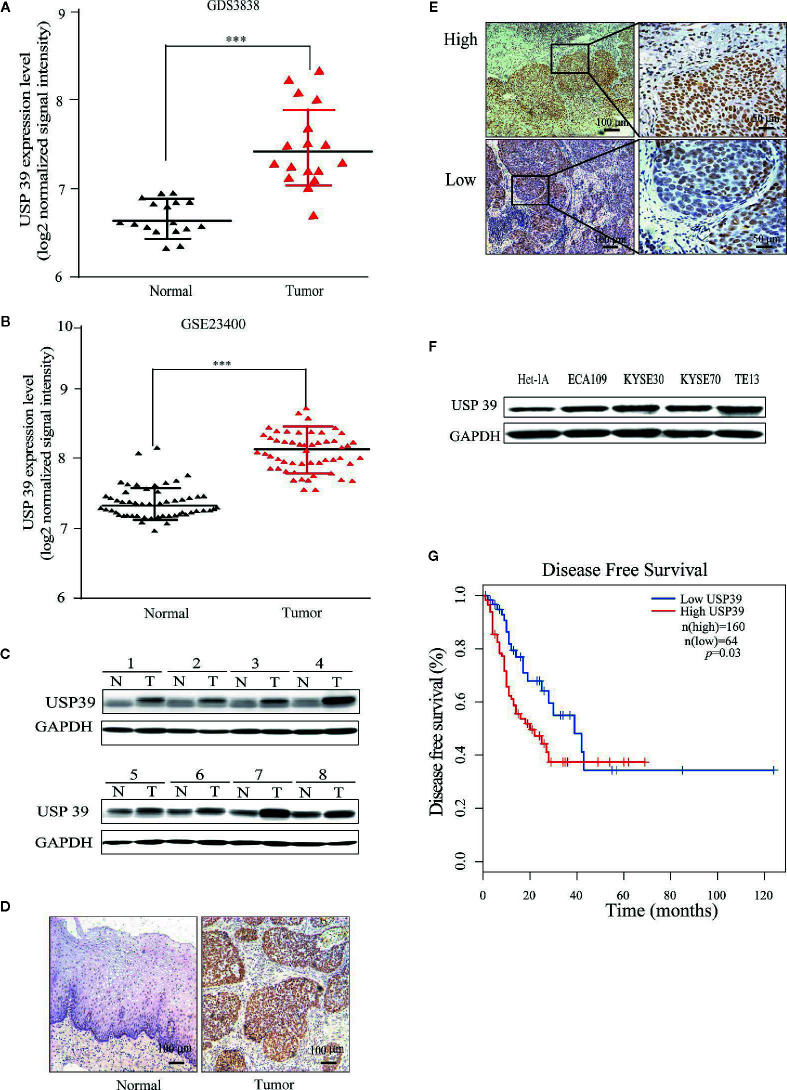
USP39 is upregulated in ESCC tissues and cell lines. **(A, B)** The relative expression of USP39 in ESCC tissues compared with normal tissues was analyzed using GEO datasets GDS3838 **(A)** and GSE23400 **(B)**. Student’s t-test: ^***^ *p* < 0.001. **(C)** Western blotting analyses of USP39 protein expression in ESCC (T) and paired adjacent normal tissues (N). USP39 protein expression levels were normalized to the GADPH loading control (n = 8 per group). **(D, E)** Representative immunohistochemical staining of USP39 expression in a matched pair of adjacent normal versus tumor tissues **(D)** and examples of high and low IRS scores in ESCC cancer tissues **(E)**. **(F)** Western blotting analyses for USP39 expression in ESCC cell lines ECA109, KYSE30, KYSE70 and TE-13 compared with normal esophageal cell line Het-1A. **(G)** Kaplan-Meier curve showing a correlation of USP39 expression with disease free survival rate in ESCC patients.

We first analyzed gene expression data from ESCC cases determined from microarray-based studies in the GEO database (GDS3838 and GSE23400). The analyses of two independent cohorts showed consistently that USP39 expression was elevated in tumor tissues compared with their paired adjacent normal tissues (**Figures 1A, B**). To verify this observation, Western blotting analyses were conducted in a small cohort of matched pairs of ESCC tissues (T) and adjacent normal tissues (N). Instructively, USP39 protein was decisively upregulated in all eight ESCC cases compared with normal tissues (**Figure 1C**). We then extended these studies to include immunohistochemical (IHC) examination of 120 pairs of ESCC samples (**Figures 1D, E**). Here strong staining for USP39 was detected in 82 (68.3%) of ESCC tissues but only in 33 (27.5%) of normal tissues (**Table 1**). Moreover, we analyzed whether the expression of USP39 was associated with ESCC clinicopathological characteristics. As shown in **Table 2**, the upregulation of USP39 in ESCC was found to be associated with tumor differentiation, invasion, lymph node metastasis and TNM stage. In contrast, other demographic factors, including age, gender and tumor location were independent of USP39 expression. We also performed Kaplan-Meier survival analysis using TCGA dataset. The results showed patient disease free survival (DFS) was significantly shorter in ESCC cases with high USP39 expression compared with low USP39 expression (**Figure 1G**), which suggests that upregulation of USP39 is correlated with the poor prognosis of ESCC. This proposes USP39 as a potential biomarker for ESCC.

**Table 1:** IHC staining of USP39 expression in ESCC tissues.

	# of cases	Expression		*P*
		Low (-/+)	High (++/+++)
Normal	120	87	33	P<0.001
ESCC	120	38	82	

P value was estimated by Chi-square test.

**Table 2:** Relationship of USP39 expression and clinicopathological characteristics in patients.

	# of cases	Expression		*P*
		Low (-/+%)	High (++/+++%)
*Age*				
≥65	72	23	49	0.936
≥65	48	15	33
*Gender*				
male	87	28	59	0.843
female	33	10	23
*Tumor location*				
Ut	19	6	13	0.950^#^
Mt	56	17	39
Lt	45	15	30
*Differentiation status*				
Well	26	21	5	<0.001
Moderate	63	15	48
Poor	31	2	29
*Invasion*				
T1+T2	47	25	22	<0.001
T3+T4	73	13	60
*Lymph node metastasis*				
Absent	69	29	40	0.005
Present	51	9	42
*TNM stage*				
I+ II	65	31	34	<0.001
III+ IV	55	7	48

Lt, lower thoracic esophagus; Mt, middle thoracic esophagus; Ut, upper thoracic esophagus.

P value was estimated by Chi-square test, ^#^P value was estimated by Mann-Whitney U test.

We also examined USP39 expression in ESCC cell lines (ECA109, KYSE30, KYSE70 and TE13) compared with normal esophageal epithelial cell line (Het-1A) using Western blotting. Consistent with the *ex vivo* analyses of ESCC tissues, the protein levels of USP39 were significantly higher in the ESCC cell lines compared with normal esophageal epithelial cells (**Figure 1F**).

### USP39 Promotes Proliferation of ESCC Cells *In Vitro*

**Figure 2:**
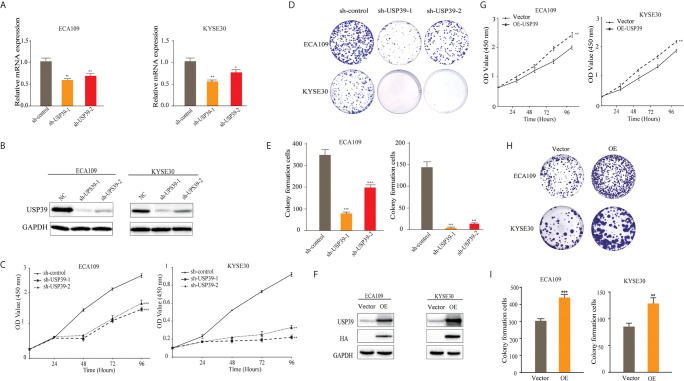
Silencing USP39 expression in ESCC cells inhibits proliferation. **(A, B)** USP39 levels were measured in the ESCC cell lines ECA109 and KYSE30 by qRT-PCR **(A)** and Western blotting **(B)** after stable knockdown with negative control shRNA or two different shRNAs targeting USP39, sh-USP39-1 and sh-USP39-2. **(C–E)** Proliferation of the cells from **(A)** was measured from 0-96 h using CCK-8 assays **(C)** or colony formation assays **(D, E)**. **(F–I)** Proliferation in vector control or USP39 overexpressing cells **(F)** was measured by CCK-8 assays **(G)** or colony formation assays **(H–I)**. The results are shown as mean ± S.D. of three independent experiments. Student’s t-test: *p < 0.05, ^**^ *p* < 0.01, ^***^ *p* < 0.001.

Based on findings in the previous Section, we sought to investigate the biological function of USP39 in ESCC cells. We first established that USP39 could be effectively suppressed in the ECA109 and KYSE30 cell lines by RNAi using independent short hairpin RNAs (shRNAs) delivered as lentiviral particles. Measurement of USP39 levels by qPCR confirmed reductions in USP39 mRNA in both cell lines with the sh-USP39-1 shRNA construct being more efficient than the other used (**Figures 2A, B**). Along with negative controls the USP39 knockdown cell lines were then subjected to cell growth measurements using both the CCK-8 and colony formation assays. Knockdown of USP39 in both ESCC cell lines resulted in decisive reductions in the ability of cells to proliferate as well as establish colonies (**Figures 2C–E**). Conversely, overexpression of USP39 in ECA109 and KYSE30 cells increased their ability to proliferate and establishing colonies compared with the vector control (**Figures 2F–I**). Together these data indicate that USP39 promotes ESCC cell proliferation *in vitro*.

### USP39 Modulates ESCC Growth *In Vivo*

**Figure 3:**
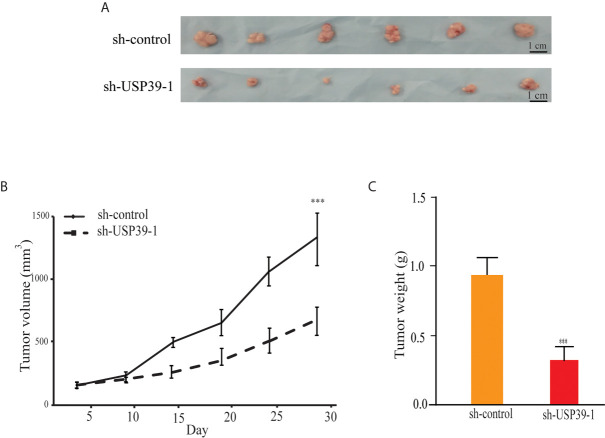
USP39 shRNA knockdown decreases xenograft growth of ESCC cells. **(A)** Representative photographs of tumor xenografts removed from nude mice at 28 days post-injection with ECA109 cells transduced with sh-control or sh-USP39-1. **(B, C)** Caliper-based measurements of tumor size measured at the indicated intervals **(B)** and final tumor weights **(C)**. The results are shown as mean ± S.D. of three independent experiments. Student’s t-test: ^***^ *p* < 0.001.

We then turned to investigate whether knockdown of USP39 also reduces ESCC cell growth in the more physiologically relevant *in vivo* context. Equal cell numbers of ECA109 cells bearing control or USP39 shRNA stable knockdown were inoculated into the flanks of nude mice and allowed to establish xenografts over four weeks. Tracking tumor growth by volume measurements showed that the growth rate of sh-USP39-1 was significantly less with the final tumor weights being consistently less than their control counterparts (**Figures 3A–C**). These results indicated that USP39 is also critical for ESCC cell proliferation and tumorigenicity *in vivo*.

### USP39 Regulates mTORC2 Signaling Through Rictor

Having established that USP39 contributes to a growth promoting phenotype in ESCC cells we sought to explore the potential mechanisms involved. We therefore conducted comparative transcriptomic analyses on control versus USP39 shRNA stable knockdown cells using RNA-seq.

**Figure 4:**
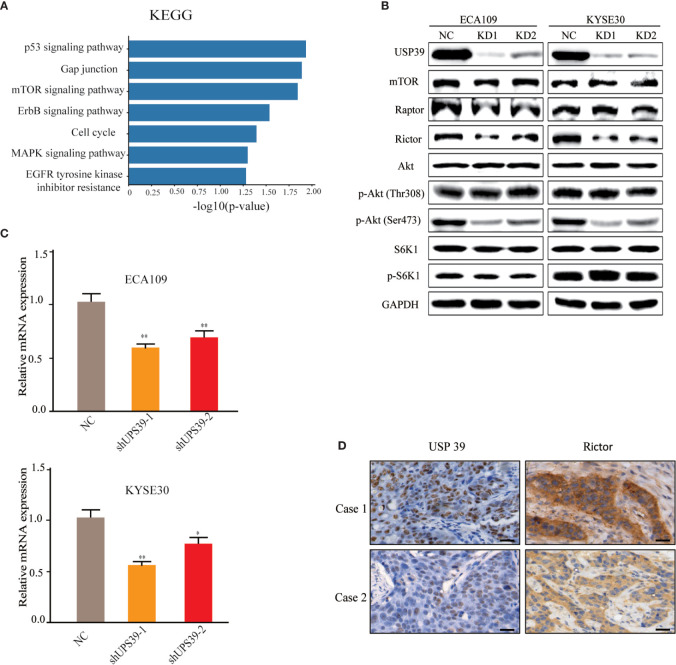
Rictor is downregulated by USP39 silencing. **(A)** RNA-seq analysis conducted on control versus USP39 shRNA-bearing ECA109 cells identified differentially regulated KEGG pathways. **(B)** Western blotting analyses of key components of mTOR signaling pathways in ECA109 and KYSE30 cells stably transduced with sh-control or sh-USP39-1, sh-USP39-2. GAPDH was used as a loading control. **(C)** qRT-PCR analyses of Rictor mRNA levels in ECA109 and KYSE30 cell lines after USP39 knockdown. The results are shown as mean ± S.D. of three independent experiments. Student’s t-test: ^*^ *p* < 0.05, ^**^ *p* < 0.01. **(D)** Representative images of IHC staining of USP39 versus Rictor in high USP39 (Case 1) and low USP39 (Case 2) IRS examples of ESCC tissues.

Analyses of differentially regulated pathways using KEGG suggested that mTOR was one of the most significant pathways altered by USP39 knockdown (**Figure 4A**). mTOR is a serine/threonine kinase that regulates cell growth and proliferation and interacts with several proteins to form two distinct complexes named mTORC1 and mTORC2, distinguished by mTORC1 containing Raptor and mTORC2 containing Rictor. Instructively, Western blotting analysis showed that the expression of Rictor, but not mTOR or Raptor, was reduced in USP39 deficient cells compared to control cells (**Figure 4B**). We further observed that USP39 knockdown reduced the phosphorylation of Akt at its hydrophobic motif (Ser473), while the phosphorylation of S6K1, a downstream target of mTORC1, exhibited little change (**Figure 4B**). These results show that targeting USP39 by RNAi inhibits mTORC2 but not mTORC1 in ESCC cells.

As a spliceosome component, we anticipated that USP39 was likely impacting Rictor expression at the mRNA level. Indeed, evaluation by qRT-PCR showed that knockdown of USP39 significantly reduced the Rictor mRNA expression in ECA109 and KYSE30 cells (**Figure 4C**). Furthermore, we performed IHC of Rictor on human ESCC samples (n = 50) and found that IHC scores for Rictor were significantly correlated with USP39 expression (p < 0.01; **Figure 4D**, **Table 3**). Thus, USP39 and Rictor are positively correlated in both ESCC cell lines and tissues. Collectively, these results demonstrated that loss of USP39 led to selective decreases in Rictor expression but did not affect other major components of the mTOR signaling pathway.

**Table 3:** Correlation of USP39 and Rictor protein expression in ESCC samples.

		USP39		*P*
		Low	High
Rictor	Low	11	6	0.005
	High	8	25

P value was estimated by Chi-square test.

**Table 4:** The primers for qRT-PCR used in this study.

	forward	reverse
USP39	5’-GGCAGTAAAACTTGAGGGTGT-3’	5’-TTGAAGTCTCACGCCTACATTC-3’
GAPDH	5’-TGACTTCAACAGCGACACCCA-3’	5’-CACCCTGTTGCTGTAGCCAAA-3’
Rictor unspliced mRNA	5’-TTGTGTGGCTTGGAGTGAGT-3’	5’-AGTTTTGCTGCACAGGTTTCA-3’
Rictor spliced mRNA	5’-AACTGGGCTTTCACTATGAGGA-3’	5’-TGGATGAGATATCGAAGCGCT-3’
S6K1 unspliced mRNA	5’-ATGAGGTCACACTGTTGCCC-3’	5’-AATTTTGGGCTGGGTGTGGT-3’
S6K1 spliced mRNA	5’-AAGAAGATGATGTCCGCCAGT-3’	5’-AGATTTCTGTGGAGGGGCAAA-3’
Rictor	5’-GGAAGCCTGTTGATGGTGAT-3’	5’-GGCAGCCTGTTTTATGGTGT-3’

### USP39 Regulates Rictor Protein Expression Through Pre-mRNA Splicing and Maturation

**Figure 5:**
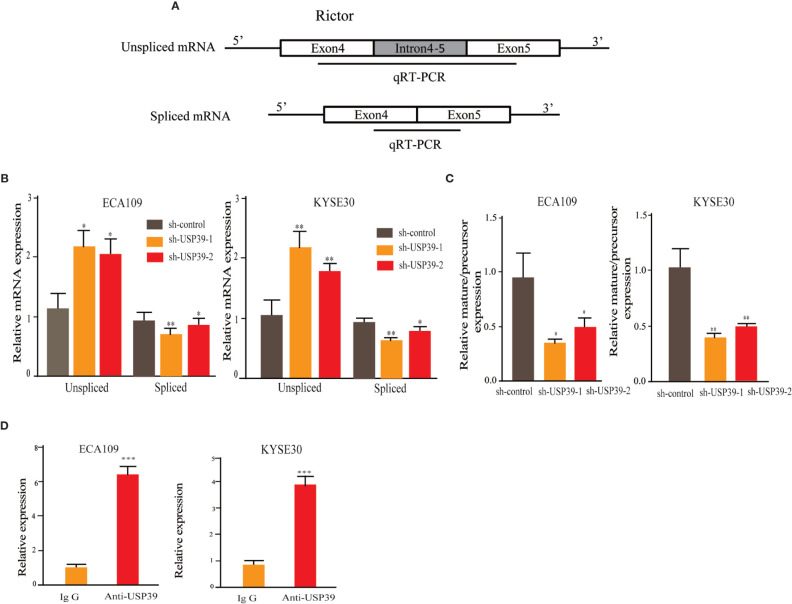
USP39 is involved in Rictor pre-mRNA splicing and maturation. **(A)** Schematic illustrating the qPCR primer pairs used to measure pre-mRNA and mature mRNA species based on the Rictor gene sequence in the Ensembl database. **(B, C)** qRT-PCR analyses showing relative mRNA levels of spliced and unspliced Rictor RNA transcripts in ECA109 and KYSE30 cells with or without USP39 shRNA knockdown **(B)**. Splicing efficiency is expressed as the ratio between mature/pre-mRNA levels **(C)**. GAPDH was used for normalization. **(D)** RNA immunoprecipitations (RIP) performed using antibodies against USP39 or an IgG control. qRT-PCR were used to measure relative Rictor mRNA levels recovered in the immunoprecipitated complex. The results are shown as mean ± S.D. of three independent experiments. Student’s t-test: ^*^ *p* < 0.05, ^**^ *p* < 0.01, ^***^ *p* < 0.001.

**Figure 6:**
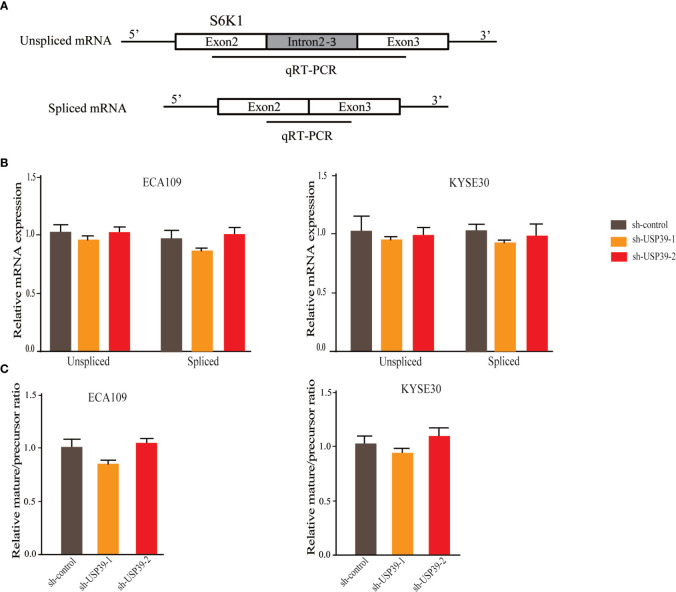
USP39 is not involved in the splicing of S6K1. **(A)** Schematic illustrating the qPCR primer pairs used to measure pre-mRNA and mature mRNA species based on the S6K1 gene sequence in the Ensembl database. **(B, C)** qRT-PCR analyses showing relative mRNA levels of spliced and unspliced S6K1 RNA transcripts in ECA109 and KYSE30 cells with or without USP39 shRNA knockdown **(B)**. Splicing efficiency is expressed as the ratio between mature/pre-mRNA levels **(C)**. GAPDH was used for normalization. The results are shown as mean ± S.D. of three independent experiments. Student’s t-test: *p* > 0.05.

Since USP39 is involved in the pre-mRNA splicing of certain genes (20, 23), we hypothesized that USP39 also regulates Rictor pre-mRNA splicing. To test this, we designed qRT-PCR primers that would distinguish the levels of unspliced pre-mRNA (intron 4-5) and mature spliced mRNA (exon 4-5 junction) (**Figure 5A**). Using this assay we found that knockdown of USP39 significantly increased the levels of unspliced mRNA transcripts compared with the controls, together with corresponding decreases in spliced mRNA (**Figure 5B**). Calculating the splicing efficiency expressed as the ratio of spliced to unspliced transcripts indicated that targeting USP39 by RNAi reduced the splicing efficiency of Rictor pre-mRNA (**Figure 5C**). In comparison, the splicing of S6K1 was not affected by USP39 knockdown (**Figure 6**).

Additionally, we performed RNA-binding protein immunoprecipitation assays (RIP) against USP39 using extracts prepared from ESCC cell lines. Analysis of the samples by qRT-PCR showed that Rictor pre-mRNA was significantly enriched in USP39 immunoprecipitates compared with IgG controls (**Figure 5D**). These results suggest that USP39 may directly regulate the splicing and maturation of Rictor pre-mRNA in ESCC cells.

## Discussion

Previous studies have reported that USP39 contributes to cancer progression and predicts poor prognosis in various human tumor types. Analyses of public datasets together with our cohort of tissues now add ESCC to the list of cancers where USP39 likely contributes to disease progression. Indeed, immunohistochemical analyses showed the upregulation of USP39 in ESCC was positively correlated with tumor differentiation status, invasion, lymph node metastasis and TNM stage. In functional experiments, we demonstrated that the upregulation of USP39 promotes cell proliferation *in vitro*, and knockdown of USP39 suppressed the tumorigenicity of ECA109 cells in a mouse xenograft model. Together these results are consistent with an oncogenic role for USP39 in the development and/or progression of ESCC.

Based on the RNA-seq analysis, we found that downstream activation of the mTOR pathway was dramatically affected by USP39 deficiency. The central element of this pathway is mTOR, a serine/threonine kinase that regulates cell growth and proliferation in response to the availability of growth factors and nutrients (26). However, mTOR exists in two functionally distinct complexes: mTORC1 and mTORC2, which have different functions. The mTORC1 complex is composed of mTOR, mLST8, PRAS40, Deptor and Raptor and enhances cell growth and proliferation through various mechanisms, whereas mTORC2 contains mTOR, mLST8, mSIN1, Deptor, Protor-1 and Rictor (27). In contrast to mTORC1, relatively little is known about the functions of mTORC2 although it has been described that mTORC2 regulates cell survival through Akt activation by S473 phosphorylation (28). Like mTORC1, recent reports also demonstrated that dysregulation of mTORC2 signaling often occurs in a variety of human malignant tumors, rendering it a crucial and validated target in cancer treatment (29–31).

The functions of mTORC1 and mTORC2 are distinguished by their differential activation of downstream effectors, mTORC1-S6K1 and mTORC2-Akt, respectively. We therefore investigated the relationship between USP39 and mTOR signaling through analysis of key mTORC1 and mTORC2 components. The protein levels of mTOR were unchanged with USP39 knockdown although there was a reduction in Rictor but not Raptor, proposing effects on mTORC2. Indeed, we found a positive correlation between USP39 and Rictor expression in our cohort using IHC. Furthermore, examining the levels and activation of their respective downstream elements, S6K1 and Akt, revealed that USP39 silencing reduced the levels of Ser473 phosphorylated Akt, but not the levels or phosphorylation status of S6K1. Interestingly, it has been previously shown that Rictor contributes to mTORC2-medicated phosphorylation of Akt in ESCC cell lines (32). Moreover, Rictor has recently been shown to be amplified in cancer, and plays an important role in cell proliferation and cell survival (33–36). Here the overexpression of Rictor has also been reported in ESCC where it positively correlated with American Joint Committee on Cancer (AJCC) disease stage and negatively impacted survival (37). Moreover, stable knockdown of Rictor inhibited the proliferation of ESCC cells *in vitro* and *in vivo* (38). Thus, our results propose that Rictor constitutes a key target of USP39 in ESCC where it plays an essential role in regulating mTORC2-mediated signaling to promote tumor cell proliferation.

**Figure 7:**
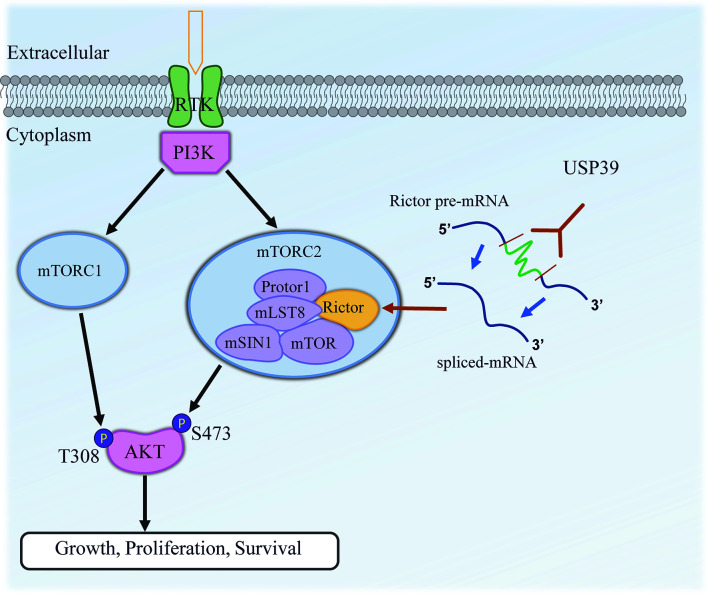
Working model illustrating the functional contribution of USP39 in regulating the activity of mTORC2 by selectively enhancing the splicing and maturation of Rictor mRNA in ESCC.

A fundamental question that remained was how USP39 drives increased Rictor expression. USP39, also known as the 65 kDa SR-related protein from early studies, has been implicated in the function of the spliceosome-complex (12). Although USP39 is a component of the U4/U6-U5 tri-snRNP, it is not necessary for tri-snRNP complex stability. Rather USP39 is necessary for spliceosome maturation and function (12). Because USP39 has been shown to be necessary for pre-mRNA splicing in particular genes including aurora B and Rb1 (14, 15), it was natural to examine for changes in Rictor mRNA splicing. One approach used to determine the splicing rate of pre-mRNA involves comparing pre-mRNA and mature mRNA transcript levels by examining the presence of transcripts containing or lacking intronic sequences (20, 23, 39). Focusing on the intron 4-5/exon 4-5 ratio we found that Rictor pre-mRNA was upregulated following USP39 knockdown, indicating a role for USP39 in the efficient maturation of Rictor mRNA. In support, we found that Rictor pre-mRNA was associated with USP39. Thus, USP39 likely regulates Rictor protein expression through effects on processing of Rictor mRNA (**Figure 7**).

## Conclusion

In conclusion, we demonstrated that USP39 is commonly upregulated in ESCC, and that it promotes malignant tumor properties in ESCC both *in vitro* and *in vivo*. Herein, USP39 appears to be an oncogenic driver in the progression of human ESCC. Functionally, we discovered a new role of USP39 in regulating mTORC2 signaling pathway due to its ability to enhance splicing and maturation of Rictor mRNA. Finally, our work provides an important basis for the development of diagnostic and therapeutic approaches in the treatment of ESCC patients by targeting USP39.

## Data Availability Statement

The original contributions presented in the study are included in the article/supplementary material, further inquiries can be directed to the corresponding author/s.

## Ethics Statement

The studies involving human participants were reviewed and approved by Anhui Medical University. The patients/participants provided their written informed consent to participate in this study. The animal study was reviewed and approved by Anhui Medical University.

## Author Contributions

XLiu, YZ and RZ designed the research. YZ, HG, GL, QJ and XC performed the experiments. YZ, HG, GL, XLi and WL analyzed the data. YZ, RZ, XLiu and RT wrote the manuscript. All authors contributed to the article and approved the submitted version.

## Funding

This work was supported by the National Natural Science Foundation of China [Grant numbers 81772908, 81201368, 31702030], and Anhui Provincial Natural Science Foundation [Grant number 1808085QH271], the Natural Science Foundation of Universities of Anhui Province [Grant number KJ2019ZD22].

## Conflict of Interest

The authors declare that the research was conducted in the absence of any commercial or financial relationships that could be construed as a potential conflict of interest.
